# Hippocampal glutamate-glutamine (Glx) in adults with Down syndrome: a preliminary study using *in vivo* proton magnetic resonance spectroscopy (^1^H MRS)

**DOI:** 10.1186/1866-1955-6-42

**Published:** 2014-11-27

**Authors:** Giles MY Tan, Felix Beacher, Eileen Daly, Jamie Horder, Verinder Prasher, Maria-Luisa Hanney, Robin Morris, Simon Lovestone, Kieran C Murphy, Andrew Simmons, Declan GM Murphy

**Affiliations:** Sackler Institute for Translational Neurodevelopment, Department of Forensic and Neurodevelopmental Sciences, Institute of Psychiatry, King’s College London, London, UK; Southern Health NHS Foundation Trust, North Hampshire Community Learning Disability Service, Winchester, Hampshire UK; Greenfields Monyhull Hospital, Kings Norton, Birmingham UK; Northumberland Tyne and Wear NHS Foundation Trust, Northgate Hospital, Morpeth, Northumberland UK; Department of Psychology, Institute of Psychiatry, King’s College London, London, UK; Department of Old Age Psychiatry, Institute of Psychiatry, King’s College London, London, UK; Department of Psychiatry, Royal College of Surgeons in Ireland, Dublin, Ireland; Department of Neuroimaging, Institute of Psychiatry, King’s College London, London, UK; NIHR Biomedical Research Centre for Mental Health and Biomedical Research Unit for Dementia, South London and Maudsley NHS Foundation Trust, London, UK

**Keywords:** Down syndrome, Intellectual disability, Alzheimer’s disease, Dementia, Magnetic resonance spectroscopy, ^1^H MRS, Hippocampus, Glutamate-glutamine (Glx)

## Abstract

**Background:**

Down syndrome (DS), or trisomy 21, is one of the most common autosomal mutations. People with DS have intellectual disability (ID) and are at significantly increased risk of developing Alzheimer’s disease (AD). The biological associates of both ID and AD in DS are poorly understood, but glutamate has been proposed to play a key role. In non-DS populations, glutamate is essential to learning and memory and glutamate-mediated excitotoxicity has been implicated in AD. However, the concentration of hippocampal glutamate in DS individuals with and without dementia has not previously been directly investigated. Proton magnetic resonance spectroscopy (^1^H MRS) can be used to measure *in vivo* the concentrations of glutamate-glutamine (Glx). The objective of the current study was to examine the hippocampal Glx concentration in non-demented DS (DS-) and demented DS (DS+) individuals.

**Methods:**

We examined 46 adults with DS (35 without dementia and 11 with dementia) and 39 healthy controls (HC) using ^1^H MRS and measured their hippocampal Glx concentrations.

**Results:**

There was no significant difference in the hippocampal Glx concentration between DS+ and DS-, or between either of the DS groups and the healthy controls. Also, within DS, there was no significant correlation between hippocampal Glx concentration and measures of overall cognitive ability. Last, a sample size calculation based on the effect sizes from this study showed that it would have required 6,257 participants to provide 80% power to detect a significant difference between the groups which would indicate that there is a very low likelihood of a type 2 error accounting for the findings in this study.

**Conclusions:**

Individuals with DS do not have clinically detectable differences in hippocampal Glx concentration. Other pathophysiological processes likely account for ID and AD in people with DS.

## Background

Down syndrome (DS) is one of the most common chromosomal disorders and is caused by trisomy 21 or a translocation involving chromosome 21 [1]. A prominent feature of DS is intellectual disability (ID) and there is an increased risk of developing Alzheimer’s disease (AD) [2]. For example, it has been estimated that the prevalence of AD in DS increases dramatically from 11% between ages 40 to 49 to as high as 77% between 60 and 69 [3–5].

The underlying cause(s) for both the ID and AD is likely to be multifactorial. For instance, it has been hypothesised that in the DS brain, the presence of an extra copy of the amyloid precursor protein (APP) gene (which is localised to chromosome 21) leads to abnormalities in amyloid precursor protein processing in neuronal membranes and subsequently to amyloid plaques and AD [6]. In addition, reduced levels of serotonin (5HT) and gamma-aminobutyric acid (GABA) have been observed in foetal DS brains [7]. Further, it has been reported that in a DS mouse model, the selective serotonin reuptake inhibitor fluoxetine normalises hippocampal neurogenesis and performance on a hippocampus-dependent memory task [8]—whereas excessive GABA-mediated inhibition impairs the induction of long-term potentiation (LTP) and memory processes in the same region [9]. We previously reported our findings of myo-inositol (mI), N-acetylaspartate (NAA), choline-containing compounds (Cho) and creatine and phosphocreatine (Cr + Pr) from the current study sample in an earlier paper which showed that adults with DS have a significantly increased hippocampal concentration of myo-inositol as compared to healthy controls [10] and that this is associated with reduced cognitive ability. Increased concentration of myo-inositol within individuals with DS may also be associated with increased risk for AD [11]. Hence, there is increasing evidence that a number of neurochemical systems likely contribute to the DS cognitive phenotype—but there have been relatively few studies on the putative role of glutamate within individuals with DS.

Normal cognitive function (including attention, learning, memory and executive processes) is supported by a number of neurotransmitters—central to which is the glutamatergic system [12]. Glutamate is the primary excitatory neurotransmitter in the brain and is involved in synaptic transmission, plasticity and excitotoxicity [13]. Enhanced glutamate release from presynaptic neurons and subsequent activation of postsynaptic alpha-amino-3-hydroxy-5-methyl-4-isoxazole-propionic acid (AMPA) and N-methyl-D-aspartate (NMDA) glutamate receptors are crucial for LTP [14, 15] and successful performance of numerous higher cognitive functions—including memory and learning [16, 17]. Also, it has been suggested that within the non-DS population, glutamate-mediated excitotoxicity contributes to the neurodegeneration and cognitive dysfunction typically observed in people with AD [18–21].

The importance of this neurotransmitter pathway in non-DS people with AD is further supported by evidence from clinical trials in these populations which showed the efficacy of drugs that target glutamatergic neurotransmission. For example, it has been reported that memantine (an NMDA receptor antagonist) reduces hippocampal glutamate concentration [22] and improves the behavioural, cognitive and functional symptoms in people with AD [23]. Therefore, there is evidence that reduction in hippocampal glutamate concentrations is associated with improved cognitive function by modulating glutamatergic neurotransmission and reducing excitotoxicity in non-DS people with AD.

Further, murine models of DS suggest 1) that there is an imbalance between hippocampal inhibitory and excitatory inputs [9, 24], 2) there are changes in the levels of the glutamate transporter and vesicular glutamate transporter 1 (VGLUT1) [25] and 3) that there are impairments in signalling mechanisms downstream of the NMDA receptor [26]. Therefore, based on the above considered evidence, it is possible that abnormalities in glutamate metabolism may partly account for *both* the ID and increased risk for AD in people with DS.

There is initial *indirect* evidence (from studies of platelets and fibroblasts) that glutamate uptake may be significantly decreased in DS individuals [27]. There have also been a small number of postmortem studies. For instance, some have reported no difference in glutamine or glutamate concentration in the frontal lobes of foetal DS brains as compared to controls [7]. In contrast, some (but not all) autopsy studies of adult DS brains reported decreased glutamate levels in the hippocampus [28] or no differences in the temporal lobes [29] or frontal lobes [30]. These studies were important first steps—but postmortem studies have inherent (and significant) limitations for measuring glutamate, and this prior work was confounded by medication effects. Also, to date, there are few studies that have directly examined *in vivo* brain glutamate in DS.

### Proton magnetic resonance spectroscopy

Proton magnetic resonance spectroscopy (^1^H MRS) can be used to measure brain concentrations of glutamate-glutamine (Glx), mI, NAA, Cho and Cr + PCr [31, 32]. In excitatory neurotransmission, glutamate is released into the synaptic cleft and is then rapidly removed by uptake into astrocytes (where it is converted into glutamine) and subsequently transported back to the presynaptic neuron for reconversion to glutamate [33, 34]. The Glx signal on ^1^H MRS can therefore be used as a measure for central glutamatergic neurotransmission—albeit without sufficient resolution to determine which particular component of the Glx cycle is abnormal.

There are only two prior ^1^H MRS studies of Glx in people with DS. These reported a significant decrease in Glx concentration within DS frontal lobe [35]—but no difference in the temporal lobe [36]. Those two studies were valuable first steps, but they only included children and did not examine brain regions most implicated in AD. Therefore, to explore the putative role that the glutamatergic neurotransmitter system plays in ID and risk of AD in DS, *in vivo* studies of adults are required.

The hippocampus may be of particular relevance in people with DS as its volume has been reported to be disproportionately reduced ( [37, 38] and see review [39]), and it is the brain region most vulnerable to the neuropathological changes of AD [40, 41]. To the best of our knowledge, there are no studies to date that have evaluated the *in vivo* concentrations of hippocampal Glx in DS adults. Therefore, given the potential contributory role of abnormalities in glutamatergic neurotransmission to both ID and AD in people with DS, we investigated the hippocampal concentration of Glx in DS adults with (DS+) and without (DS-) dementia using ^1^H MRS.

## Methods

### Participants

We included 85 adults: 46 DS individuals (35 DS- and 11 DS+) and 39 healthy controls (Table 1). Individuals with DS were recruited from cohorts in London, Birmingham and Newcastle upon Tyne, England. Karyotyping was used to assess the DS status in all participants. Dementia status was assessed using *International Statistical Classification of Diseases, 10th Revision* research criteria [42].

**Table 1:** **Demographic, MRI and** ^**1**^ **H MRS characteristics by group**

	Non-demented DS (DS-)	Demented DS (DS+)	Healthy controls (HC)	Significance
*N* = 85	*n* = 35	*n* = 11	*n* = 39	
Demographics, mean (percent)				
Male no.	26 (74%)	6 (55%)	24 (62%)	*p* = 0.358^a^
Age, mean (SD)	35 (12)	52 (6)	35 (12)	*p* < 0.001^b^
Cognitive measures				
CAMCOG (total) score	56 (22)	32 (21)	119 (3)	*p* < 0.001^b^
CAMCOG (short-term memory) score	12 (7)	5 (4)	22 (2)	*p* < 0.001^b^
MRI VOI proportions				
Grey proportion (average R and L)	0.76 (0.07)	0.75 (0.11)	0.72 (0.08)	*p* = 0.187^b^
White proportion (average R and L)	0.24 (0.07)	0.25 (0.11)	0.28 (0.08)	*p* = 0.187^b^
Mean CSF (R)	0.13 (0.07)	0.15 (0.04)	0.07 (0.04)	*p* = 0.000^b^
Mean CSF (L)	0.10 (0.07)	0.17 (0.09)	0.07 (0.04)	*p* = 0.006^b^
Mean metabolite concentration				
Glx (R hippocampus)	38.89 (6.13)	41.04 (4.16)	38.49 (7.03)	*p* = 0.309^c^
Glx (L hippocampus)	36.51 (5.25)	36.56 (7.20)	36.59 (5.72)	*p* = 0.955^c^
Glx (average of R and L)	38.14 (5.61)	38.41 (4.71)	37.57 (5.16)	*p* = 0.853^c^

*L* left, *R* right, *CAMCOG* Cambridge Cognitive Examination, *VOI* volume of interest.

^a^Chi-square.

^b^ANOVA.

^c^ANCOVA (covaried with age).

All participants underwent standard physical, neurological and psychiatric screening, including routine blood tests (e.g. renal and liver function tests, red blood cell count and thyroid function tests) and clinical magnetic resonance imaging. We excluded people with a clinically detectable physical or psychiatric disorder affecting brain function (e.g. hypertension), a known history of birth trauma or head injury, or with an abnormal clinical magnetic resonance image (for example, as indicated by the presence of significant white matter hyperintensities). None of the participants were taking psychotropic medication at the time of the study. The project was approved by Multi-Centre Research Ethics Committee (MREC) and Local Research Ethics Committees (LREC), and after complete description of the study to the participants and their identified carers, written informed consent was obtained from them or if this was not possible, assent was obtained from their identified carers.

### ^1^H MRS data acquisition and analysis

#### ^1^H MRS protocol

The subjects were scanned using a 1.5 Tesla GE NV/i Signa MR System (General Electric, Milwaukee, WI, USA) at the Maudsley Hospital, London. 3D T1-weighted volume images were acquired in the axial plane with 1.5-mm contiguous sections using acquisition parameters chosen using a contrast simulation tool [43]. Repetition time (TR) was 13.8 ms, inversion time (TI) 450 ms, echo time (TE) 2.8 ms and the flip angle was 20° with one data average and a 256 × 256 × 124 voxel matrix. Acquisition time was 6 min, 27 s.

**Figure 1:**
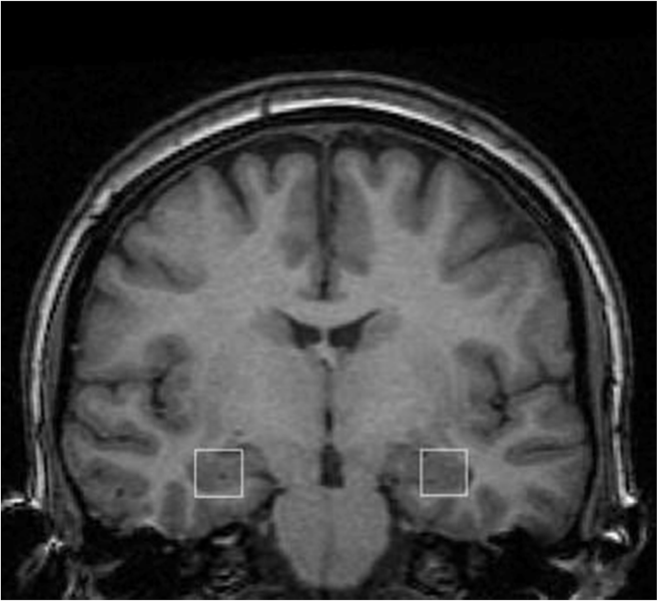
**Coronal T1-weighted magnetic resonance image illustrating the location of the** ^**1**^ **H MRS voxels in the left and right hippocampi.**

^1^H MRS voxels of interest measuring 20 × 20 × 15 mm^3^ (6 ml) were defined in standard locations in the left and right hippocampi using a previously published method [44, 45]. We chose hippocampal regions of interest as they were of particular relevance in DS and one of the earliest sites of change in AD. The anterior location of the voxel was defined as the coronal slice where the amygdala disappeared, extending posteriorly 20 mm and so covering the bulk of the hippocampus (Figure 1). The hippocampal volume of interest contained both grey and white matter and included some superior medial portions of the parahippocampal gyrus and the posterior portion of the amygdala.

A point resolved spectroscopy (PRESS) pulse sequence (TE 35 ms, TR 1500 ms, 256 data averages and 2,048 points) with automated shimming and water suppression and excellent reproducibility [46] was used to obtain spectra from each voxel after CHESS water suppression with high signal-to-noise ratio and clearly resolved NAA, Cho, mI, Cr + PCr and Glx peaks among other metabolites. Non-water-suppressed data were also collected for water referencing, but data was not collected to measure metabolite T1 and T2 relaxation times for individual subjects due to the limited tolerance of DS subjects for MRI scanning. Results are expressed as relaxation time corrected ratios to unsuppressed water. Not all subjects had spectral data from both left and right hippocampi. No significant differences were found in the metabolite content between the right and the left side of the hippocampus. Therefore, we averaged the metabolite measures from the left and right hippocampi from the subjects which had data from both hemispheres.

#### ^1^H MRS data analysis

Differences in proportions of white and grey matter in the ^*1*^*H MRS* voxels may confound group differences in metabolite concentrations. Thus, to ensure that differences in tissue composition did not account for metabolic differences between subject groups, we segmented the SPGR volumes using Statistical Parametric Mapping (SPM) software (http://www.fil.ion.ucl.ac.uk/spm) to determine the percentage of grey matter, white matter and CSF within the MRS voxels after quality control of the images as described previously [47, 48]. The position of the ^*1*^*H MRS* voxels relative to the segmented 3D dataset was determined automatically using an in-house software. T1 and T2 corrections were applied for each metabolite using literature values [49].

Spectra were processed using LCModel [50], and metabolite concentrations were automatically corrected for CSF contamination of the voxel by dividing by the tissue fraction of the MRS voxel determined using SPM. These corrected concentrations were then calibrated to absolute molar units with respect to a phantom of known concentration, which was scanned in the same scanning session as the subject, using a PRESS acquisition with the same TE and TR.

### Cognitive assessment

Cognitive ability was measured using the Cambridge Cognitive Examination (CAMCOG) [51, 52]. The CAMCOG has been validated for use with adults with DS [53] and provides a measure of general cognitive function, including measures of episodic memory (which is associated with hippocampal function), orientation, language, attention, praxis and executive function. The CAMCOG, developed originally to measure cognitive functioning in people with mild to moderate dementia, is less subject to floor effects and so found to be also appropriate for people with DS. For each participant, neuropsychological testing was completed within 6 months of scanning.

### Statistical analysis

Statistical analysis was carried out using SPSS (SPSS 18.0 for Windows; SPSS Inc., Chicago, IL, USA). Comparisons between age and ^1^H MRS Glx concentrations between the groups were made using univariate general linear models (GLM). Differences in gender distribution were tested for using a chi-squared test. Group differences in Glx concentrations were tested with one-way analysis of variance (ANOVA), with group as the between-subject factor. There were no significant interactions between the side from which ^*1*^*H MRS* Glx concentrations were measured (left or right hippocampus) or gender and group. To verify that voxel composition was not obscuring group differences, we performed an ANOVA with corrected Glx as the dependent variable, Group (DS-, DS+, HC) and Side (left vs. right) as a between-subject factor and covariates being voxel grey, white and CSF proportion. In this analysis, neither Group nor Group × Side were significant predictors of estimated Glx (*p* = 0.199 and *p* = 0.550) indicating that even controlling for voxel composition, there was no group difference in Glx in either left or right hippocampus. Therefore, mean hippocampal Glx concentrations were considered in the analysis with age used as a covariate.

For each of the groups, the relationship between hippocampal Glx concentrations and cognitive ability and memory (measured by the CAMCOG total and short-term memory scores) was examined using Pearson’s product–moment correlation. Level of statistical significance was defined as *p* < 0.05 (two tailed). Power calculations were performed using the mean Glx concentration differences between the groups in this study on G*Power version 3.1.5.

## Results

**Figure 2:**
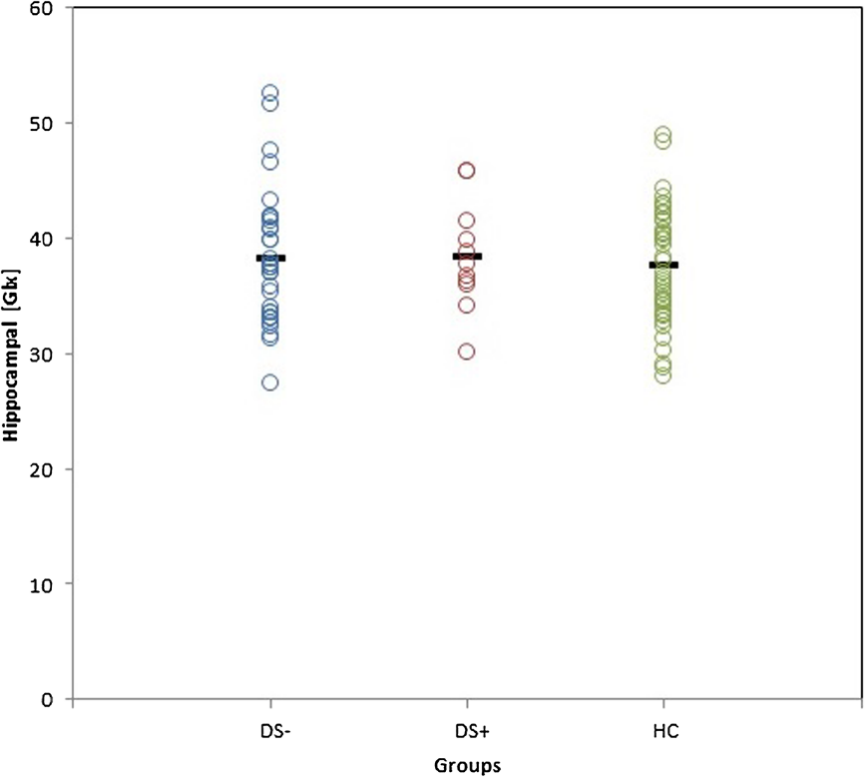
**Scatter plot of average Glx concentrations of both hippocampi for all groups.** No significant differences across groups (*p* = 0.853). *Glx* glutamate-glutamine, *DS-* non-demented Down syndrome subjects, *DS+* demented Down syndrome subjects, *HC* healthy controls. Note: horizontal bars represent the means for each group.

The results are summarised in Table 1 and Figure 2. The DS+ group were older than both the DS- and healthy control groups. Therefore, age was added as a covariate in the analyses. As expected, the groups also differed in their CAMCOG (total cognition and short-term memory) scores—with the demented DS+ group having the lowest scores and the HC having the highest.

Grey and white matter composition of the MRS voxels did not differ between the groups, although there were differences between the groups for CSF composition. Overall, we found no significance between group differences in Glx concentrations. Furthermore, there was no correlation between hippocampal Glx concentration and cognitive ability and memory as measured by the CAMCOG (total and short-term memory) scores in either of the DS groups (see Table 2).

**Table 2:** **Correlation of cognitive measures with mean hippocampal Glx concentrations for each group**

	Non-demented DS (DS-)	Demented DS (DS+)
CAMCOG (total) score	*r* = 0.13 (*p* = 0.464)	*r* = -0.225 (*p* = 0.715)
CAMCOG (short-term memory) score	*r* = 0.123 (*p* = 0.497)	*r* = -0.296 (*p* = 0.569)

Due to our relatively small sample size of DS+, it is possible that we were underpowered to detect differences. Hence, we carried out a power analysis. This showed that it would have required 6,257 participants to provide 80% power to detect a significant difference in Glx between the groups at *p* < 0.05 which would indicate a very low likelihood of a type 2 error accounting for our findings.

## Discussion

### Hippocampal Glx in DS and when compared with the general population

We found no significant between-group differences in hippocampal Glx concentration. The Glx findings in our study are consistent with (and extends into demented individuals) 1) an earlier ^1^H MRS study of DS children that reported no differences in temporal lobe Glx concentration [36] and 2) postmortem studies of adult DS brains that had not detected any increase in temporal lobe glutamate [29]. Hence, our findings suggest that the hippocampal glutamatergic neurotransmitter system (at least as measured using Glx at 1.5 Tesla) is not significantly dysregulated in people with DS—and so is not the main cause for the ID (or AD) typically found in the disorder.

Other factors, therefore, most likely play a greater role. These may include dysregulation of acetylcholine (ACh) [54], dopamine [55], GABA [56] and serotonin (5-HT) [57]; and/or brain metabolites such as mI [10]. ACh has long been known to be a critical mediator of learning and memory [58], and cholinergic neurons are particularly affected in AD [54]. Dopamine [55] and GABA modulate memory, and drugs that act as inverse agonists at the GABA(A) receptor have shown promise in enhancing memory function [56]. mI is elevated in the hippocampus of DS- adults and is negatively correlated with their cognitive ability [10] and further increased in DS+ individuals [11].

### Hippocampal Glx in demented DS+ versus AD in the general population

In this study, we did not find any differences in hippocampal Glx concentration between DS+ individuals when compared to either their DS- counterparts or healthy controls. The DS+ group were significantly older than the DS- and HC groups.

To the best of our knowledge, there are no previous studies that have examined hippocampal Glx in people with DS and AD. Our findings (albeit in a limited sample) are consistent with some (but not all) ^1^H MRS studies of AD in the general population. For instance, some reported no differences in the Glx concentration of temporoparietal grey matter between AD individuals and healthy controls [59], whereas others reported decreased glutamate in the right hippocampus [60]. Thus, the hippocampal metabolic changes in people with DS and AD may differ from those of non-DS people with AD.

However, our initial evidence taken together with the work of others suggests that the situation may be different in people with DS. For instance, two recent randomised double-blind placebo-controlled trials which investigated the efficacy of memantine in DS people found limited effect of treatment with memantine on cognitive or functional outcomes. Boada et al. compared the effect of 16-week treatment with either memantine or placebo on cognitive and adaptive functions of 40 young adults with DS and found no significant differences between the memantine and placebo groups on the two primary outcome measures involving episodic memory [61]. In the study by Hanney et al. which compared adults with DS with and without dementia and involved 88 patients receiving memantine and 85 patients receiving placebo showed that both groups declined in cognitive and functional ability but rates did not differ between groups for any cognitive or functional outcomes at 52 weeks of treatment [62]. Therefore, in people with DS, dysregulation of glutamatergic neurotransmission and related glutamate-mediated excitotoxicity may not be the major pathway for the development of either ID or AD. This has implications for the development of new treatments.

Various risk factors and hypotheses for the pathogenesis of AD in DS have been proposed [63] including the role of β-amyloid accumulation in the DS brain [6]. However, a recent *in vivo* study using positron emission tomography (PET) showed that DS- individuals have comparable concentrations of β-amyloid in the brain as compared with non-DS people with AD [64] and would suggest that there are factors other than β-amyloid loading which are important for the development of AD in people with DS.

These other factors may include apolipoprotein E ϵ4 allele [65], extended tau haplotype [66], dual-specificity tyrosine-regulated kinase 1A (DYRK1A) and calcipressin [67], tetranucleotide repeat in intron 7 of the APP [68], estradiol [69], Cu/Zn superoxide dismutase (SOD1) [70], neuroinflammation [71] and serotonergic dysfunction [8, 72]. In particular, mI which has been associated with dementia in the general population [73] and may also have particular relevance in people with DS as the Na^+^/myo-inositol co-transporter gene (SCL5A3) located on chromosome 21 [74] has been shown in our previous work to be increased in the hippocampus of DS- [10] and further increased in DS+ [11] may point to a potential role of mI in the cascade of events that lead to AD in people with DS.

#### Limitations

Our study had a relatively small sample size, and we included relatively few demented DS+ participants. However, due to the small differences between the groups, it would have required an unrealistically large number of participants in the study to show an effect. A sample size calculation based on the effect sizes from this study showed that it would have required 6,257 participants to provide 80% power to detect a significant difference between the groups. People with DS are a difficult population to recruit to studies, and they all have ID and can have difficulty tolerating the demands of the scanning procedure and cognitive assessments. Furthermore, the participants in our study were not medicated or sedated prior to their brain scans to facilitate the scan procedure.

The study was conducted at relatively low field strength (1.5 T), and glutamate was expressed as Glx rather than as glutamate and glutamine separately. Glutamate, glutamine and GABA exist in a metabolite shuttle known as the glutamate/GABA-glutamine cycle. Measuring Glx on its own may not have detected changes in the metabolite shuttle as GABA could not be accurately measured at low field strength. It is possible that Glx metabolite changes may have occurred in brain regions that were not examined in this study, but the hippocampus was chosen due to its known involvement in memory and early involvement in AD. We did not examine other brain regions critical to higher cognitive function owing to limitations in patient compliance and time constraints. Despite that, we managed to scan a sizeable number of non-demented DS participants in this study and did not find any differences in Glx concentration compared to healthy controls. We have previously reported differences in myo-inositol concentration in a similar number of participants which would indicate that even if there were differences in Glx concentrations between the groups, these differences are much less pronounced than those affecting myo-inositol.

Future studies involving larger numbers of DS+ participants, using higher field strength ^1^H MRS and using multi-voxel ^1^H MRS approaches will allow better spectral segregation of glutamate and glutamine and measurement of GABA as well as the examination of other regions of the brain critical to higher cognitive function.

## Conclusions

We found no evidence that DS individuals (with or without dementia) have significant dysregulation of glutamatergic neurotransmission. Other factors may be more crucial in the development of both ID and AD in people with DS.
